# Baseline right bundle branch block and clinical outcomes in patients undergoing transcatheter aortic valve implantation: a Danish nationwide cohort study

**DOI:** 10.1093/ehjopen/oeag004

**Published:** 2026-01-17

**Authors:** Daniel K Steiner, Maria L Krogager, Christoffer Polcwiartek, Claus Graff, Helle C Christensen, Mikkel P Andersen, Christian J Terkelsen, Evald H Christiansen, Jens C Nielsen, Mads B Kronborg, Anders M Sommer, Troels Thim, Julia Ellert, Jens B Johansen, Gintautas Bieliauskas, Ole De Backer, Michael Vinther, Carsten Simonsen, Sam Riahi, Signe J Riddersholm, Christian Torp-Pedersen, Kristian Hay Kragholm, Ashkan Eftekhari

**Affiliations:** Department of Cardiology, Aalborg University Hospital, Hobrovej 18-22, 9000 Aalborg, Denmark; Department of Cardiothoracic Surgery, Aalborg University Hospital, Hobrovej 18-22, 9000 Aalborg, Denmark; Department of Cardiology, Aalborg University Hospital, Hobrovej 18-22, 9000 Aalborg, Denmark; Department of Cardiology, Regional Hospital of Northern Jutland, Bispensgade 37, 9800 Hjørring, Denmark; Department of Cardiology, Aalborg University Hospital, Hobrovej 18-22, 9000 Aalborg, Denmark; Aalborg University, Department of Science Health and Technology, Selma Lagerløfs Vej 249, 9260 Gistrup, Denmark; The Prehospital Center, University Hospital of Copenhagen, Ringstedsgade 61, 4700 Naestved, Denmark; Department of Cardiology, Nordsjællands Hospital, Dyrehavevej 29, 3400 Hillerød, Denmark; Department of Cardiology, Aarhus University Hospital, Palle Juul-Jensens Boulevard 99, 8200 Aarhus N, Denmark; Department of Cardiology, Aarhus University Hospital, Palle Juul-Jensens Boulevard 99, 8200 Aarhus N, Denmark; Department of Cardiology, Aarhus University Hospital, Palle Juul-Jensens Boulevard 99, 8200 Aarhus N, Denmark; Department of Cardiology, Aarhus University Hospital, Palle Juul-Jensens Boulevard 99, 8200 Aarhus N, Denmark; Department of Cardiology, Aarhus University Hospital, Palle Juul-Jensens Boulevard 99, 8200 Aarhus N, Denmark; Department of Cardiology, Aarhus University Hospital, Palle Juul-Jensens Boulevard 99, 8200 Aarhus N, Denmark; Department of Cardiology, Odense University Hospital, J.B. Winsløwsvej 4, 5000 Odense C, Denmark; Department of Cardiology, Odense University Hospital, J.B. Winsløwsvej 4, 5000 Odense C, Denmark; Copenhagen Heart Center, Blegdamsvej 9, 2100 Copenhagen, Denmark; Copenhagen Heart Center, Blegdamsvej 9, 2100 Copenhagen, Denmark; Copenhagen Heart Center, Blegdamsvej 9, 2100 Copenhagen, Denmark; Department of Cardiothoracic Surgery, Aalborg University Hospital, Hobrovej 18-22, 9000 Aalborg, Denmark; Department of Cardiology, Aalborg University Hospital, Hobrovej 18-22, 9000 Aalborg, Denmark; Department of Cardiology, Aalborg University Hospital, Hobrovej 18-22, 9000 Aalborg, Denmark; Department of Cardiology, Nordsjællands Hospital, Dyrehavevej 29, 3400 Hillerød, Denmark; Department of Public Health, University of Copenhagen, Øster Farimagsgade 5, 1353 København K, Denmark; Department of Cardiology, Aalborg University Hospital, Hobrovej 18-22, 9000 Aalborg, Denmark; Department of Cardiology, Aalborg University Hospital, Hobrovej 18-22, 9000 Aalborg, Denmark

**Keywords:** Transcatheter aortic valve replacement, Right bundle branch block, Pacemaker, Heart failure, Electrocardiogram

## Abstract

**Aims:**

In the largest nationwide study of Danish patients undergoing transcatheter aortic valve implantation, we examined the clinical impact of pre-existing right bundle branch block – a known risk factor for permanent pacemaker implantation – on outcomes including pacemaker implantation, heart failure, and all-cause mortality.

**Methods and results:**

We included first-time transcatheter aortic valve implantation patients in Denmark from 2008 to 2021. Patients were stratified by baseline right bundle branch block status using the digital Danish Nationwide Electrocardiogram Cohort. The study outcomes were new pacemaker implantation, heart failure, and all-cause mortality at 30, 90, and 365 days. A composite of the study outcomes was also assessed. A total of 4900 patients were included, of whom 438 (9%) had baseline right bundle branch block. Mean age was 81 years, 55% were male. At 90 days, the overall pacemaker implantation incidence was 9%, but markedly higher in the right bundle branch block group: 28.3% vs. 7.1% [hazard ratio (HR) 4.61, 95% confidence interval (CI) 3.60–5.91]. Right bundle branch block was not associated with higher rates of heart failure (10.1% vs. 8.3%; HR 1.23, 95% CI 0.86–1.77) or death (3.7% vs. 3.6%; HR 1.08, 95% CI 0.64–1.82). The findings were consistent at 1 year.

**Conclusion:**

In this comprehensive nationwide cohort, right bundle branch block strongly predicted pacemaker implantation after transcatheter aortic valve implantation but importantly, not heart failure or all-cause mortality. The neutral association with heart failure and mortality should be explored further.

## Graphical Abstract

**Graphical Abstract:**
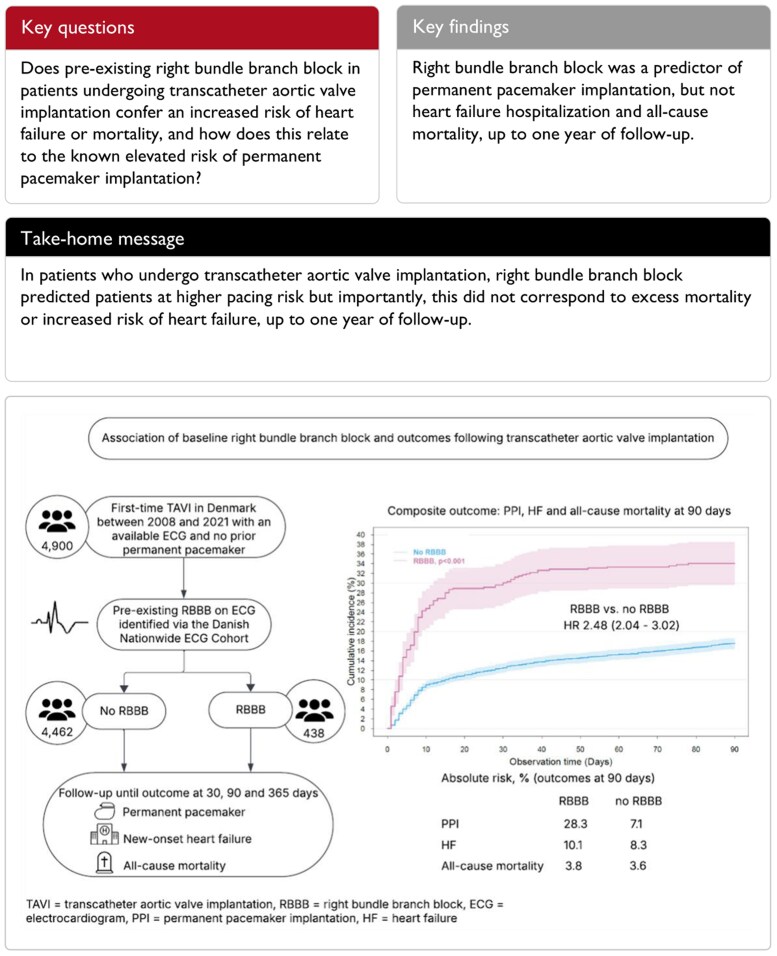

## Introduction

Aortic stenosis is the most common type of primary valvular heart disease requiring intervention in Europe and North America.^1^ Transcatheter aortic valve implantation (TAVI) has become standard therapy in patients over 70 years of age or high risk for surgical aortic valve replacement (SAVR).^1^ Furthermore, TAVI use in low-risk patients is increasing following landmark trials that expanded use to these patient groups.^2,3^ Although early heart-failure (HF) readmission remains a concern after TAVI, recent randomized trials and nationwide registry analyses suggest that contemporary TAVI does not confer a higher short-term HF burden than SAVR.^2–4^ Right bundle branch block (RBBB) has previously been established as a predictor of atrioventricular (AV) block with need for subsequent permanent pacemaker implantation (PPI) in patients undergoing TAVI.^5–7^ The increased risk of severe conduction disturbances has led to some centres implementing a prophylactic PPI strategy in patients with pre-existing RBBB.^8^

Although RBBB has been associated with increased risk of PPI, there are currently no official guideline recommendations on the timing of PPI in this specific patient population.^8^ Moreover, there are no data from randomized controlled trials in patients with RBBB undergoing TAVI.

Using the Danish Nationwide Electrocardiogram (ECG) Cohort and Danish health registries, the objective of this nationwide study was to investigate the incidence of new PPI, HF, and all-cause mortality in a cohort of Danish patients with aortic stenosis undergoing TAVI from 2008 to 2021, stratified by pre-TAVI RBBB status.

## Methods

### Study population

This was a Danish nationwide register-based cohort study. All patients with an available ECG within 180 days before TAVI and a diagnosis of aortic stenosis who underwent first-time TAVI in Denmark between 1 January 2008 and 31 December 2021 were included in the study. All access sites were included (i.e. transfemoral, transapical, or transaortic).

### Data source

All Danish residents are assigned a unique personal identification number at birth or upon immigration. This identification number can be used in all health-care contacts and links data such as in- and outpatient hospital diagnoses [classified according to the International Classification of Diseases, Tenth Revision (ICD-10)] and surgical/interventional procedures [classified according to the NOMESCO Classification of Surgical Procedures (NCSP)] to the Danish National Patient Register.^9^ Information on prescription drugs based on the Anatomical Therapeutic Chemical Classification (ATC) was obtained from the Danish National Prescription Register.^10^ Information on patient sex, age, vital status, and emigration was obtained from the Civil Registration System.^11,12^ The Danish Nationwide ECG Cohort contains information on standard 12-lead digital ECG recordings. Recordings come from pre-hospital or in-hospital settings, containing information such as personal identification number, date and time of acquisition, and highly detailed ECG parameters including heart rate, QRS duration, *P*-wave duration, PR-interval, QT interval, frontal axis of QRS, *P* and T, ST-segment elevation at the J-point, and waveform amplitudes. ECGs are analysed using the Marquette 12 SL algorithm resulting in diagnostic statements such as RBBB, third-degree AV block, and atrial fibrillation.^13^ The use of the 12 SL algorithm to diagnose RBBB has been favourably validated with high specificity (ranging from 99.8% to 100%) and sensitivity (ranging from 90% to 93.2%) when compared to diagnosis by a cardiologist using a traditional definition of RBBB.^14^

The exclusion criteria were PPI before TAVI. PPIs were identified via relevant NCSP codes (BFCA01-07 and BFCA6), including single- and dual-chamber as well as biventricular pacemakers. Aortic stenosis was identified using the TAVI NCSP-codes (KFMD11-13), since aortic stenosis is the only indication for TAVI in Denmark.

Relevant comorbidities and baseline medications were identified using ICD-10 codes or ATC codes as appropriate (see *Table 1* for full details). Hypertension was defined as at least two anti-hypertensive drug prescriptions within 90 days.^15^ Lipid lowers, antidiabetics, and inhalation drugs were identified by relevant ATC codes within 180 days before study inclusion.

**Table 1:** Baseline characteristics

	No RBBB (*n* = 4462)	RBBB (*n* = 438)	Total (*n* = 4900)
Males	2404 (53.9)	304 (69.4)	2708 (55.3)
Age, years, median [IQR]	81 [76, 85]	82 [78, 85]	81 [77, 85]
**ECG characteristics, *n* (%)**			
Atrial fibrillation/flutter	928 (20.8)	57 (13.0)	985 (20.1)
Left bundle branch block	795 (17.8)	0 (0.0)	795 (16.2)
First-degree AV block	656 (14.7)	98 (22.4)	754 (15.4)
Second-degree AV block, type I	7 (0.2)	NA	NA
Second-degree AV block, type II	9 (0.2)	NA	NA
Second-degree AV block, 2:1 conduction	6 (0.1)	NA	NA
Left anterior hemiblock	123 (2.8)	119 (27.2)	242 (4.9)
Left posterior hemiblock	7 (0.2)	13 (3.0)	20 (0.4)
**Comorbidities, *n* (%)**			
Ischemic heart disease	2208 (49.5)	250 (57.1)	2458 (50.2)
Myocardial infarction	785 (17.6)	87 (19.9)	872 (17.8)
Stroke	633 (14.2)	60 (13.7)	693 (14.1)
Diabetes mellitus	906 (20.3)	109 (24.9)	1015 (20.7)
Hyperlipidemia	1608 (36.0)	162 (37.0)	1770 (36.1)
Hypertension	2737 (61.3)	276 (63.0)	3013 (61.5)
Chronic obstructive pulm. Disease	744 (16.7)	80 (18.3)	824 (16.8)
Chronic kidney disease	432 (9.7)	60 (13.7)	492 (10.0)
Peripheral arterial disease	590 (13.2)	56 (12.8)	646 (13.2)
Heart failure	1247 (27.9)	126 (28.8)	1373 (28.0)
**Comedications, *n* (%)**			
Lipid lowers	330 (7.4)	30 (6.8)	360 (7.3)
Antidiabetics	121 (2.7)	18 (4.1)	139 (2.8)
Antihypertensive medications	135 (3.0)	15 (3.4)	150 (3.1)
**Procedures, *n* (%)**			
Percutaneous coronary intervention	1122 (25.1)	118 (26.9)	1240 (25.3)
Coronary artery bypass graft	414 (9.3)	54 (12.3)	468 (9.6)
Ablation therapy (any type)	66 (1.5)	NA	NA
Mitral valve surgery	37 (0.8)	NA	NA

IQR, interquartile range; NA, denotes observations ≤ 3 and ≥ 1 which are considered microdata and therefore required to be censored.

### Exposure, outcomes, and follow-up

The exposure was an RBBB diagnostic statement identified using the Marquette 12 SL algorithm. The outcomes of interest were risk of PPI and new-onset HF (both with death as a competing risk) as well as all-cause mortality at 30, 90, and 365 days. A composite of these outcomes at the same time points was also included in the analysis. An HF diagnosis was identified using the ICD-10 codes DI110, DI130, DI132, DI42, and DI50. For this outcome, two separate analyses were made: In the first analysis, patients with an HF diagnosis before TAVI were included. An outcome diagnosis of HF was interpreted as worsening HF or readmission. In the second analysis, patients with a HF diagnosis before TAVI were excluded, and an outcome diagnosis of HF was interpreted as *de novo*/incident HF. Patients were followed up to 1 year from TAVI to an outcome of interest, death, or emigration, whichever occurred first.

### Statistics

Percentages and number counts are reported for categorical data, and continuous data are reported with means and standard deviation (SD). Aalen–Johansen estimates for the primary events, as well as the competing risk of death by RBBB exposure status, were used for cumulative incidence curves. Cause-specific Cox regressions were performed to analyse all outcomes, where death was accounted for as a competing risk, in relation to whether RBBB was present at the latest ECG before the TAVI procedure. All statistical inferential tests were two-sided at the 0.05 significance level. All Cox regression assumptions were tested for interactions, linearity, and proportionality of hazards. Interactions were tested for age, sex, and left anterior fascicular block. Cox model assumption tests are shown in Supplementary material online, *Figures S1*  &  *S2*. Cox models for outcomes of interest and the competing risk of death were used to derive models to predict outcomes at 30 and 90 days. These were used in G-estimation to calculate outcomes standardized to the whole population.^16^

Data management and statistical analyses were performed using SAS, version 9.4 (SAS Institute Inc., Cary, NC, USA) and R, version 4.0.3 (R Foundation for Statistical Computing, Vienna, Austria).

### Sensitivity analyses

Two separate sensitivity analyses were performed for the study: (1) An analysis including additional adjustment for continuous QRS duration (scaled per 10 ms). Since a wider QRS generally reflects greater underlying His-Purkinje disease, we included this analysis to evaluate how the degree of QRS widening may influence the study outcomes. Before modelling, this continuous variable was evaluated by dividing QRS duration into quartiles and fitting a Cox model with Q1 as reference. Analyses were repeated for all outcomes at 30, 90, and 365 days (PPI, HF, and all-cause mortality). (2) An analysis with patients stratified by the presence of bifascicular block (i.e. RBBB and left anterior/posterior hemiblock), RBBB and first-degree AV block, or bifascicular block and first-degree AV block. Cause-specific Cox regression was performed for these exposures to determine whether the risk of PPI differed when considering these combined conduction abnormalities rather than RBBB alone. This was done to assess whether an increased risk associated with RBBB was driven primarily by isolated RBBB or by the presence of concomitant conduction disturbances.

### Ethics

The use of the data sources applied for the conduct of this study was approved by the data responsible institute in the Capital Region of Denmark. In accordance with Danish legislation studies that are conducted for the sole purpose of statistical and scientific research do not require ethical approval.^17^

## Results

**Figure 1:**
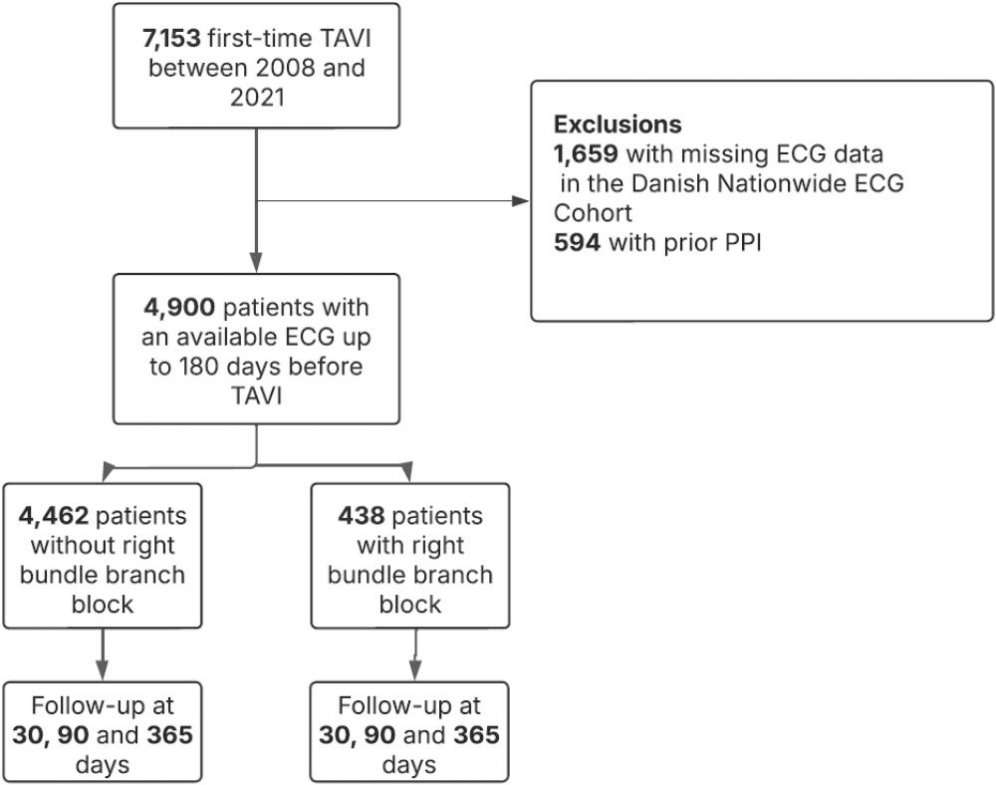
Cohort selection flowchart.

A total of 4900 first-time TAVI patients were included in the study after exclusion of 2253 patients (see *Figure 1* for details). 55% were male and the mean age of patients was 81 years. A total of 438 patients (9%) had a pre-existing RBBB on ECG before TAVI. Transfemoral access was used in 90% of cases, 6% transapically and 4% via transaortic access. Baseline characteristics are shown in *Table 1*.

### Outcomes

**Figure 2:**
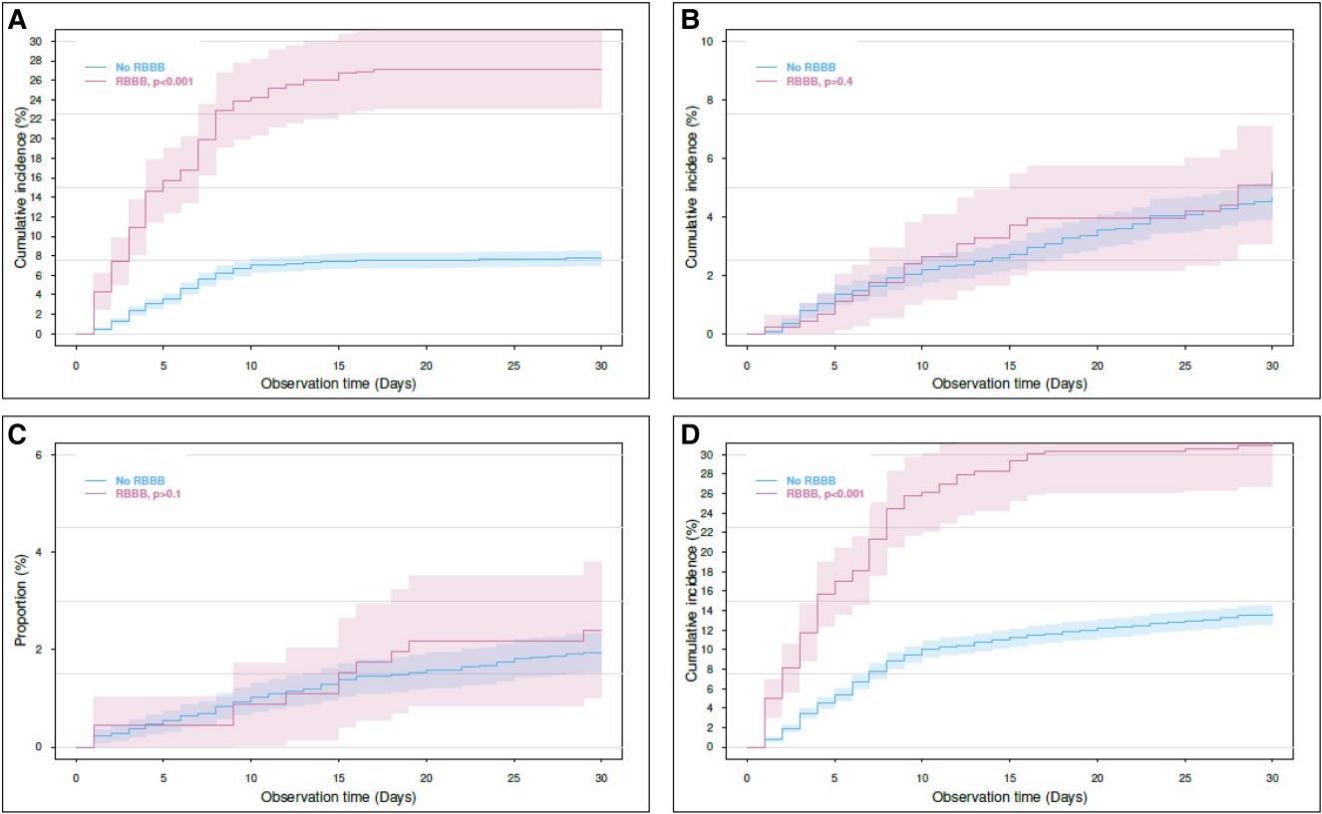
Cumulative incidence curves for permanent pacemaker implantation, heart failure, all-cause mortality, and composite outcome at 30 days. Panel A: Permanent pacemaker implantation. Panel B: New-onset heart failure. Panel C: All-cause mortality. Panel D: Composite of all three outcomes. Pre-existing RBBB was associated with a significantly higher rate of permanent pacemaker implantation, but not heart failure and death at 30 days. Significance level 0.05.

Of the entire study population, a total of 397 (8%), 427 (9%), and 491 (10%) patients required a PPI at 30 days, 90 days, and 1 year post TAVI, respectively. At those same time points, 223 (5%), 397 (8%), and 694 (14%) had a diagnosis of HF post TAVI. All-cause mortality at 30 and 90 days was 2% vs. 4%, respectively. All-cause mortality at 1 year was 10%. Cumulative incidence curves at 30 days are shown in *Figure 2*.

**Figure 3:**
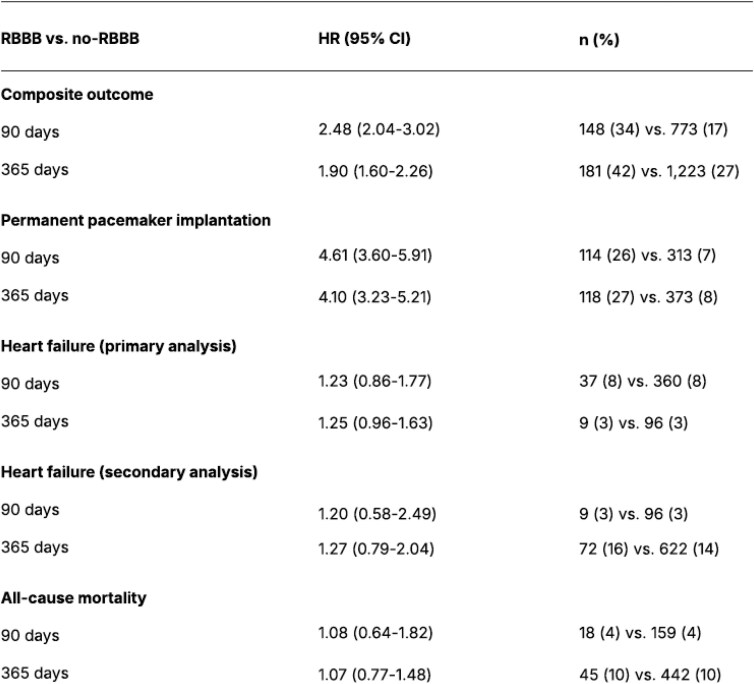
Hazard ratios (multivariable model) and absolute numbers for the outcomes at 90 and 365 days (95% CI). HR, hazard ratio; CI, confidence interval; *n*, number of patients. Pre-existing RBBB was associated with a significantly higher rate of permanent pacemaker implantation, but not heart failure (primary and secondary analysis) and death at 90 and 365 days. Heart failure (HF) primary analysis: Patients with pre-existing HF were included in the analysis. HF secondary analysis: Patients with pre-existing HF were excluded from the analysis. Significance level 0.05.

Pre-existing RBBB was associated with a significantly higher rate of the composite outcome at 30 days: Hazard ratio (HR) 3.13 at 95% confidence interval [95% confidence interval (CI) 2.53–3.87]. Likewise, pre-existing RBBB was associated with a significantly higher rate of PPI at 30 days post TAVI: HR 4.96 (3.85–6.40). At 30 days post TAVI, 111 (25%) vs. 286 (6%) patients had a PPI (RBBB vs. no-RBBB). Pre-existing RBBB was not associated with an increased risk of HF. HF outcome (primary analysis): In this analysis, patients with an HF diagnosis before TAVI were included: The 30-day HR was 1.49 (0.94–2.37). At 30 days post TAVI, 24 (6%) vs. 199 (5%) patients had a diagnosis of HF (RBBB vs. no-RBBB). HF outcome (secondary analysis): In this secondary analysis, patients with an HF diagnosis before TAVI were excluded and a total of 3527 patients were included in this analysis, 312 of which had pre-existing RBBB. A pre-existing RBBB was not associated with a significantly higher rate of the outcome at 30 days: HR 2.07 (0.88–4.88). At 30 days post TAVI 7, (2%) vs. 49 (2%) had an HF diagnosis in this secondary analysis (RBBB vs. no-RBBB). Pre-existing RBBB was not associated with an excess mortality risk at 30 days: HR 1.39 (0.72–2.71). At 30 days, 11 (3%) vs. 87 (2%) patients had died (RBBB vs. no-RBBB). *Figure 3* presents the hazard ratios for all outcomes at 90 and 365 days. At 90 and 365 days, pre-existing RBBB was associated with a higher risk of PPI, but not HF and all-cause mortality.

The absolute risk, risk difference and average risk ratio for the outcomes at 30 and 90 days is shown in *Table 2*.

**Table 2:** Average treatment effect

	Absolute risk %, (95% CI)	Risk difference %, (95% CI)	*P*-value	Average risk ratio %, (95% CI)	*P*-value
**PPI at 30 days**					
No RBBB	6.4 (6–7)				
RBBB	27.8 (23–33)	21.3 (16–26)	<0.001	4.32 (3.39–5.24)	<0.001
**HF at 30 days**					
No RBBB	4.5 (4–5)				
RBBB	6.6 (4–9)	2.0 (−1–5)	0.14	1.46 (0.83–2.10)	0.16
**All-cause mortality at 30 days**					
No RBBB	1.9 (1–2)				
RBBB	2.6 (1–4)	0.6 (−1–2)	0.42	1.36 (0.47–2.25)	0.43
**PPI at 90 days**					
No RBBB	7.1 (6–8)				
RBBB	28.3 (23–33)	21.3 (16–26)	<0.001	4.01 (3.18–4.84)	<0.001
**HF at 90 days**					
No RBBB	8.3 (8–9)				
RBBB	10.1 (7–13)	1.7 (−2–5)	0.32	1.21 (0.81–1.62)	0.30
**All-cause mortality at 90 days**					
No RBBB	3.6 (3–4)				
RBBB	3.8 (2–5)	0.2 (−1–2)	0.78	1.07 (0.58–1.55)	0.78

Average treatment effect (ATE): The estimated mean difference in outcome between individuals assigned to the treatment and those assigned to the control condition in the target population, representing the expected effect of the intervention if applied to all eligible subjects.

### Sensitivity analysis

**Figure 4:**
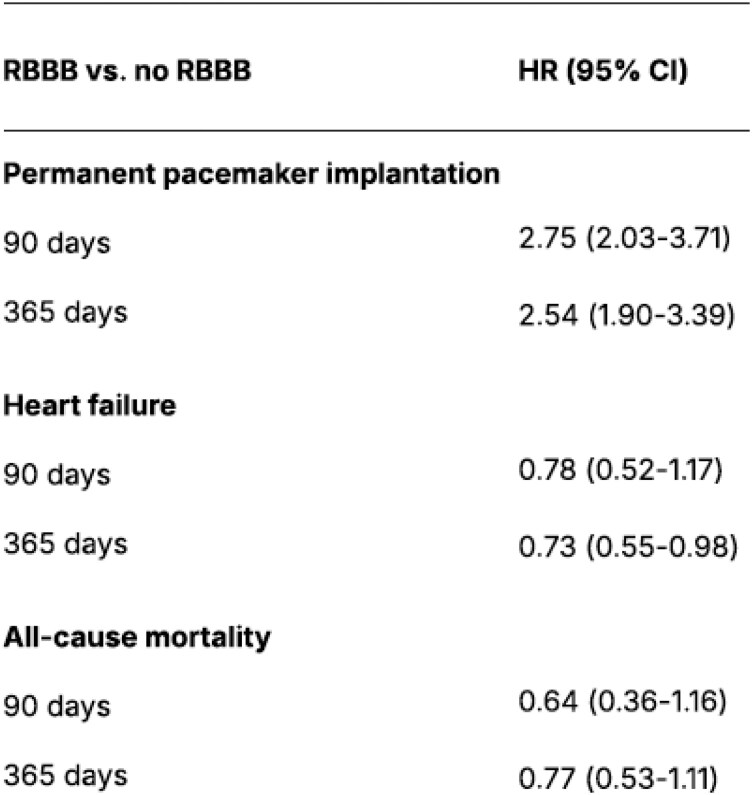
Hazard ratios (multivariable model) for the cox models adjusted by continuous QRS duration (scaled per 10 ms). Outcomes are presented at 90 and 365 days (95% CI). HR, hazard ratio; CI, confidence interval. Attenuation of the association between baseline RBBB and PPI was observed, suggesting that some part of the observed risk reflects the degree of underlying intraventricular conduction delay rather than the RBBB alone.

When QRS duration (per 10 ms) was added to the multivariable models, the association between RBBB and PPI was attenuated across all time points, although RBBB remained independently associated with 30−, 90−, and 365-day PPI. At 30 days, the HR was 2.86 (2.10–3.90). In contrast, for HF and all-cause mortality, additional adjustment for QRS duration resulted in only minimal attenuation of the HRs, and RBBB remained unassociated with either outcome at all examined time points. The 30-day HF HR in the adjusted model was 0.92 (0.55–1.55). The 30-day all-cause mortality HR was 0.87 (0.41–1.88). *Figure 4* presents the hazard ratios for the adjusted outcomes at 90 and 365 days. In the adjusted models including QRS duration, the global proportional hazards test was acceptable (*P* = 0.074). The formal test suggested some time-dependence for the effect of QRS duration (*P* = 0.009), but inspection of residual plots did not indicate a major deviation from proportionality. The correlation between RBBB and QRS duration was moderate (*r* = 0.35).

**Figure 5:**
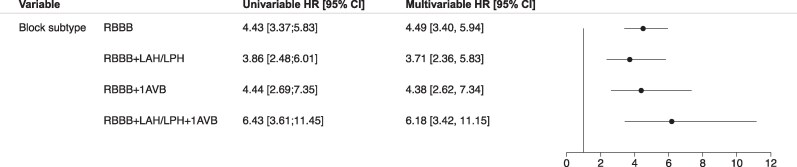
Risk of permanent pacemaker implantation at 30 days with patients stratified based on the presence of bifascicular block, RBBB, and first-degree AV block, or bifascicular block and first-degree AV block. LAH, left anterior hemiblock; LPH, left posterior hemiblock; 1AVB, first-degree AV block; HR, hazard ratio; CI, confidence interval. Patients with all block subtypes had significantly higher rates of permanent pacemaker implantation at 30 days.

*Figure 5* presents the HR for PPI at 30 days with patients stratified based on the presence of (1) bifascicular block, (2) RBBB and first-degree AV block or (3) bifascicular block and first-degree AV block rather than RBBB alone. Patients with all block subtypes had significantly higher rates of PPI at 30 days: HR 3.71 (2.36–5.83) for patients with bifascicular block, 4.38 (2.62–7.34) for patients with RBBB and first-degree AV block, and 6.18 (3.42–11.15) for patients with bifascicular block and first-degree AV block.

## Discussion

In this nationwide cohort of Danish first-time TAVI, pre-existing RBBB was strongly associated with an increased risk of PPI but not HF and all-cause mortality, up to 1 year of follow-up. This study corroborates the well-established fact that baseline RBBB is a strong predictor of PPI post-TAVI. However, the findings of this study also indicate that the presence of pre-existing RBBB does not translate into excess mortality or increased risk of HF in short- or intermediate-term follow-up.

This neutral association with HF and mortality is a clinically meaningful and less explored outcome in the TAVI literature. Few studies have investigated the role of baseline RBBB on outcomes and results of these are mixed.^18–20^ The results of our study mirror those of Weferling et al.^18^ showing that RBBB pre-TAVI is not associated with increased short-term mortality, despite much higher PPI rates in the RBBB group. The study by Weferling et al.^18^ included 1871 TAVI patients, 190 (10%) of which had pre-existing RBBB, and the PPI rates at 30 days were 46% vs. 13% in the RBBB vs. no-RBBB groups, respectively. To our knowledge, the study by Weferling et al.^18^ is the most contemporary study of patients with baseline RBBB pre-TAVI, but the study does not report on new-onset HF. In another cohort of 3527 patients, 362 (10%) of which had pre-existing RBBB, Auffret et al.^20^ reported significantly higher 30-day PPI rates (40%) and rates of all-cause mortality (10%), when compared to patients without RBBB. However, this study reports on outcomes between 2010 and 2017, and thus reflects earlier eras of device iteration and procedural techniques. Advancements in procedural techniques likely mitigate some of the adverse effects on AV conduction attributed to RBBB. For instance, high implantation techniques have been associated with a reduced incidence of new-onset conduction disturbances in newer iterations of transcatheter heart valves.^21^ In a contemporary register-based study of 4847 first-time TAVI patients in Denmark between 2010 and 2021, a statistically significant and temporally decreasing trend in all-cause mortality, HF readmission and PPI over time was observed.^4^ The 30-day mortality rate of ∼2% reported by Lundahl et al.^4^ in the latter end of follow-up, is in line with the findings of this study, although the 30-day HF readmission rate of ∼9% is higher than the 5% reported in our study. All in all, it is plausible that the improvement in TAVI outcomes over time is a result of improved valve designs and implantation techniques, growing operator experience as well as improved patient selection.

The findings of this study also relate to the ongoing discussion around prophylactic pacemaker implantation in patients with pre-existing RBBB. In this cohort, a substantial proportion of patients with RBBB did not undergo PPI during follow-up, suggesting that many individuals with RBBB may complete the post-procedural course without requiring permanent pacing. This pattern may support consideration of a more selective approach, incorporating structured rhythm monitoring, extended telemetry when appropriate, and individualized assessment rather than a strategy of prophylactic implantation. As newer-generation valves and contemporary implantation techniques appear to be associated with lower rates of conduction disturbances, the potential benefit of routine prophylactic PPI in patients with RBBB, may be less clear in current practice. These observations align with a strategy that emphasizes careful monitoring, deferred decision-making and close monitoring before deciding on PPI.

In the sensitivity analysis, adjustment for continuous QRS duration substantially attenuated the association between baseline RBBB and PPI, suggesting that some part of the observed risk reflects the degree of underlying intraventricular conduction delay rather than the RBBB alone. Nonetheless, RBBB remained associated with PPI after adjustment, indicating that both the binary diagnosis and continuous conduction measures carry complementary prognostic information. Importantly, this attenuation did not extend to the HF or all-cause mortality outcomes, for which baseline RBBB remained unassociated with both outcomes after adjustment for QRS duration.

## Limitations

There are several limitations to this study that must be acknowledged. First, this was an observational study; thus, it is beyond the scope of this study to draw causal inferences or provide explanations for the observed outcomes. Second, a major limitation to this study is that information on valve type (self-expandable vs. balloon-expandable), valve size, annulus calcification, and implantation depth were not available. These are known factors associated with conduction disturbances, and their absence introduces residual confounding and limits interpretability.^7,22^ Also, seeing that PPI risk varied widely, both in the clinical trials, and in the studies discussed above, information on valve type would have provided a useful stratification of outcomes in this study. Third, the diagnosis of HF in the Danish National Patient Register has been validated with a 76% positive predictive value.^17^ The two separate analyses (pre-TAVI HF included or excluded) were intended to allow for interpretation of *de novo*/incident HF vs. worsening HF, but some uncertainty regarding this outcome exists. Finally, of all 427 patients who received a permanent pacemaker within 90 days post TAVI identified via pacemaker procedure codes, only 28% of these had a corresponding diagnosis code. 25% of these were high grade AV block (defined as Mobitz type II, 2:1 or higher conduction block and 3rd degree AV block) and 3% were due to sinus node dysfunction. This very low diagnostic coding completeness likely reflects that clinicians often use a diagnosis code that simply states that the patient was implanted with a pacemaker, instead of coding the actual indication.

However, this study still provides valuable information in the setting of a large, nationwide cohort over a 13-year period, particularly with regards to the neutral association with HF and all-cause mortality in patients with pre-existing RBBB, which is still a subject with heterogenous outcomes in the published literature. It is the largest population-based, digital ECG dataset that can be linked to register-based data. The diagnostic statements on the ECG provided by the used algorithm have high accuracy and validity.^13^

## Conclusion

In this nationwide register-based study on 4900 first-time TAVI patients in Denmark from 2008 to 2021, pre-existing RBBB was a predictor of PPI, but not HF and all-cause mortality, at short-term follow-up of 30 and 90 days and intermediate-term follow-up at 1 year.

## Supplementary Material

oeag004_Supplementary_Data

## Data Availability

The data used in this study are securely stored on servers maintained by Statistics Denmark. They are not available for distribution in accordance with Statistics Denmark regulations. Access to the servers and the associated datasets may be granted by Statistics Denmark, contingent upon the acquisition of appropriate authorization.
