# Supplementary figures and images for: Baseline right bundle branch block and clinical outcomes in patients undergoing transcatheter aortic valve implantation: a Danish nationwide cohort study
Source: Eur Heart J Open. 2026 Jan 17;6(1):oeag004. doi: 10.1093/ehjopen/oeag004 (PMC12891907; doi:10.1093/ehjopen/oeag004)

**
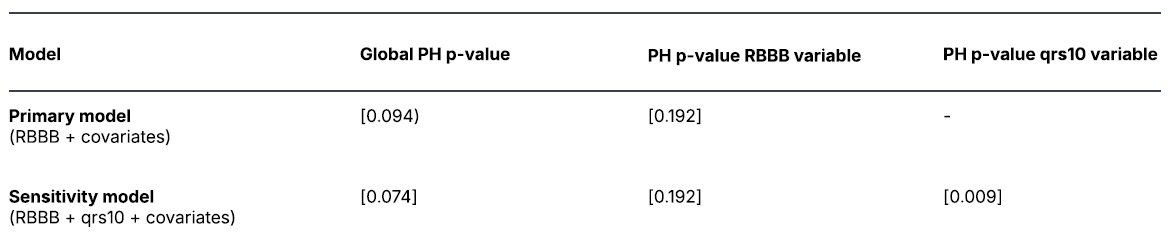
**

Supplement: oeag004_Supplementary_Data [file oeag004_supplementary_data.zip › Supplementary Figure 1.docx]

**
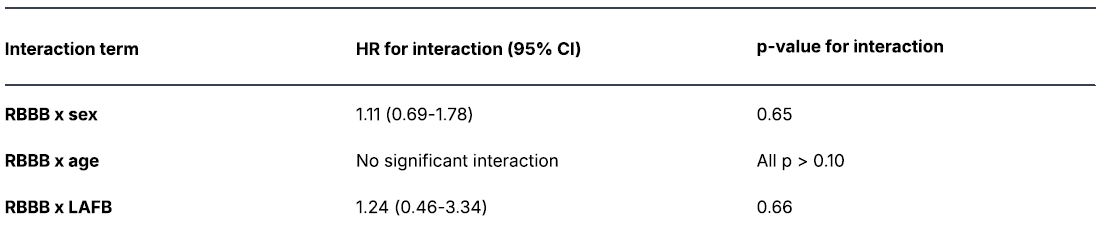
**

Supplement: oeag004_Supplementary_Data [file oeag004_supplementary_data.zip › Supplementary Figure 2.docx]
